# The Impact of Wearing Different Face Masks on Vigorous Physical Exercise Performance and Perceived Exertion among COVID-19 Infected vs. Uninfected Female Students

**DOI:** 10.3390/ejihpe13110187

**Published:** 2023-11-15

**Authors:** Nourhen Mezghani, Achraf Ammar, Omar Boukhris, Liwa Masmoudi, Mohamed Ali Boujelbane, Rayda Ben Ayed, Turki Mohsen Alzahrani, Atyh Hadadi, Rihab Abid, Ibrahim Ouergui, Jordan M. Glenn, Khaled Trabelsi, Hamdi Chtourou

**Affiliations:** 1 Department of Sport Sciences, College of Education, Taif University, Taif 21944, Saudi Arabia; mezghanni.nourhen@yahoo.fr (N.M.); tm.alzahrani@tu.edu.sa (T.M.A.); atyhhadadi@gmail.com (A.H.); 2 Department of Training and Movement Science, Institute of Sport Science, Johannes Gutenberg-University Mainz, 55099 Mainz, Germany; dbouja@yahoo.com; 3 Interdisciplinary Laboratory in Neurosciences, Physiology and Psychology: Physical Activity, Health and Learning (LINP2), UFR STAPS (Faculty of Sport Sciences), UPL, Paris Nanterre University, 39200 Nanterre, France; 4 High Institute of Sport and Physical Education of Sfax, University of Sfax, Sfax 3000, Tunisia; liwa.masmoudi@yahoo.fr (L.M.); trabelsikhaled@gmail.com (K.T.); h_chtourou@yahoo.fr (H.C.); 5 Research Laboratory, Molecular Bases of Human Pathology, LR19ES13, Faculty of Medicine, University of Sfax, Sfax 3029, Tunisia; isseps.rihababid@gmail.com; 6 SIESTA Research Group, School of Allied Health, Human Services and Sport, La Trobe University, Melbourne 3086, Australia; omarboukhris24@yahoo.com; 7 Sport, Performance, and Nutrition Research Group, School of Allied Health, Human Services and Sport, La Trobe University, Melbourne 3086, Australia; 8 Research Laboratory, Education, Motricity, Sport and Health (EM2S), LR15JS01, High Institute of Sport and Physical Education of Sfax, University of Sfax, Sfax 3000, Tunisia; 9 Physical Activity, Sport, and Health, UR18JS01, National Observatory of Sport, Tunis 1003, Tunisia; 10 National Institute of Agronomy of Tunisia (INAT), University of Carthage-Tunis, 43 Avenue Charles Nicolle, El Mahrajène 1082, Tunisia; raydabenayed@yahoo.fr; 11 Laboratory of Extremophile Plants, Centre of Biotechnology of Borj-Cédria, B.P. 901, Hammam Lif 2050, Tunisia; 12 High Institute of Sport and Physical Education of Kef, University of Jendouba, El Kef 7100, Tunisia; ouergui.brahim@yahoo.fr; 13 Research Unit, Sports Science, Health and Movement, University of Jendouba, El Kef 7100, Tunisia; 14 Department of Health, Exercise Science Research Center Human Performance and Recreation, University of Arkansas, Fayetteville, AR 72701, USA; jordan@neurotrack.com

**Keywords:** SARS, post-infection, exercise, physical activity, surgical mask, N95, performance, intermittent exercise, student, sport science

## Abstract

Under certain circumstances, masks are an effective and immediate solution to reduce the spread of viral infection. However, the impact of masks on the ability to perform vigorous exercise remains an area of concern. Primarily, this impact has been explored in healthy subjects, yielding contradictory findings, and little is known of it among COVID-19-infected individuals. This study examined the effects of surgical masks, N-95 masks, and unmasked conditions on the performance and perceived exertion (RPE) of infected vs. non-infected young women during high-intensity, repeated sprint exercise (5mSRT). Following a familiarization session, eighty-three (42 COVID-19-previously infected (PIG) and 43 non-infected (NIG)), female participants (age 20.02 ± 1.05 years, BMI 21.07 ± 2.1 kg/m^2^) were randomly assigned to one of three mask conditions: unmasked, surgical mask, or N95 mask. All participants attended three test sessions (i.e., one session for each mask condition) at least one week apart. At the beginning of each test session, data related to participants’ physical activity (PA) and sleep behaviours during the previous week were collected. In each test session, participants performed the 5mSRT, during which performance indicators (best distance (BD), total distance (TD), fatigue index (FI) and percentage decrement (PD)) were collected, along with RPE. ANOVA indicated no significant main effects of Groups and Masks, and no significant interaction for Groups × Masks for BD, FI, PD, RPE and most sleep and PA behaviours (*p* > 0.05). For TD, the Groups × Mask interaction was significant (*p* = 0.031 and ƞp^2^ = 0.042). Posthoc analysis revealed, in the unmasked condition, there was no difference in TD between PIG and NIG (*p* > 0.05). However, when wearing a surgical mask, PIG covered lower TD compared to NIG (*p* < 0.05). Additionally, different types of masks did not affect TD in NIG, while PIG performed the worst using the surgical mask (*p* < 0.05). These results suggest post-COVID-19 individuals can maintain physical fitness through regular exercise (i.e., sport science curricula) in unmasked conditions, but not when wearing a surgical mask. Furthermore, the impact of different types of face masks on physical performance seems to be minimal, particularly in uninfected populations; future research is warranted to further explore this impact in post-COVID conditions.

## 1. Introduction

According to the World Health Organization (WHO), as of 1 September 2023, more than 770 million confirmed cases of COVID-19 had been identified, resulting in approximately seven million deaths. Additionally, more than 13.5 billion vaccine doses have been administered worldwide [1].

Recent research indicates the majority of COVID-19 cases worldwide develop the long-COVID syndrome [2]. Long COVID encompasses multiple adverse effects, with new common pathologies such as cardiovascular, thrombotic, and cerebrovascular disease [3], where symptoms can last for years [4,5]. Long COVID is associated with all age groups and all forms of acute disease severity [2]. Most patients with long-onset COVID were not hospitalised at the time of their COVID-19 initial infection [2], and factors found to be associated with a higher risk of long COVID include identifying as female [6]. Long COVID is an area of ongoing research, with many open questions, particularly with regard to preventive measures likely to enhance individuals’ immunity, while simultaneously reducing the risk of infection. The WHO recommends revising old strategies against the disease, such as avoiding crowds in open spaces, wearing masks, and opening windows in enclosed indoor spaces, even for those who are vaccinated. The aim is to reduce both individual infection risk and the risk of spreading the virus to others [7].

In conjunction, masks reduce the spread of influenza and severe acute respiratory syndrome (SARS). Surgical masks and N95 respirators are equally effective in preventing transmission and limiting disease spread [8], thereby controlling and containing infectious outbreaks [9,10]. As a result, wearing a mask is still an immediate solution, assistive in all circumstances of risk for protection from any subsequent viral pandemic [11].

**Figure 1:**
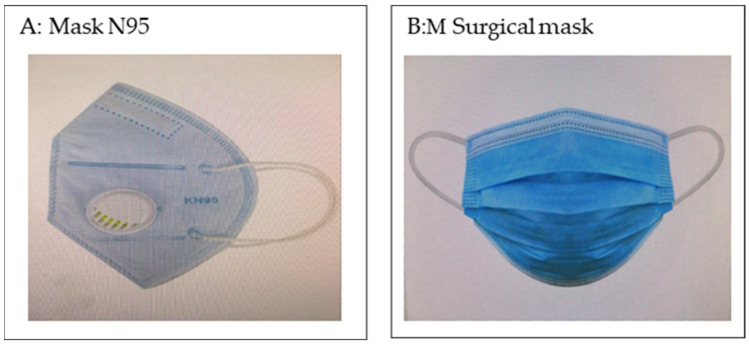
Mask type used in the experiment.

The surgical mask (Figure 1A) is a disposable, high-quality medical mask providing protection against the release of both large and small airborne particles. However, the edges of this type of mask may not create a completely airtight seal, allowing for some air leakage. N95 masks (Figure 1B), when used with respirators, are specifically designed to filter out dangerous airborne particles. They are engineered to fit snugly around the face, preventing air leaks. Prior research suggests N95 respirators offer higher degrees of protection, resulting in fewer observed respiratory and influenza-like illnesses, compared to surgical options [12,13]. Currently, numerous different standards exist worldwide, delineating testing procedures for essential factors, including fluid resistance, breathability, bacterial filtration efficiency (BFE), particle filtration efficiency (PFE), and other parameters crucial for assessing the quality of face masks [14,15]. This underscores the significance of studying the effects of various masks, as an enduring and critical subject of research.

Engaging in daily physical activity can reduce the risk of developing most chronic diseases, consequently decreasing the likelihood of mortality from COVID-19 [16]. Exercise has been shown to boost immune function [17] and is especially effective at improving the immunological response to SARS-CoV-2 antigens [18]. The literature suggests that adapted and supervised physical training may serve as an effective multisystemic therapy for post-COVID-19 syndrome [17,19]. Engaging in regular exercise within a safe environment is a crucial strategy for maintaining a healthy lifestyle during the transitional phase after the crisis, while also mitigating the potential risk associated with the emergence of new, more transmissible sub-variants [20,21]. Furthermore, physical activity has the potential to increase endorphin, dopamine, and serotonin levels, countering the negative psychological and metabolic effects of quarantine and the pandemic [22]. Consequently, it is advisable for both post-COVID and uninfected individuals to incorporate physical exercise into their routines to promote overall well-being [23].

Regarding exercise, it is recommended to include high-intensity training (HIT) and sprint-interval training (SIT) as part of the medical management plan for patients with chronic diseases due to their potential beneficial impacts. HIT, in particular, has a specific molecular signature demonstrating its ability to improve both immunological function and physical performance [24].

Due to the potential for viral transmission during high-intensity exercise, where respiratory droplets may travel farther due to forceful breathing [25], concerns have arisen regarding indoor exercise settings, especially in gyms [26] and fitness centers [27,28]. As a result, masking may become an essential component of physical activity during periods of increased disease transmission. However, there is limited and controversial information about the effects of different types of facemasks on physiological function during high-intensity exercise [21,29].

Recent studies raise concerns that use of masks during exercise may increase resistance to inspiration and breathing, as well as carbon dioxide rebreathing, resulting in hypercapnic hypoxia and decreased tissue oxygenation [30]; these issues may reduce exercise capabilities. However, others suggest mask use has no significant impact on exercise performance [11]. In this context, Epstein et al. [31] found no significant difference in time to exhaustion when wearing surgical or N-95 masks compared to being unmasked during a progressive cycle ergometer test. Nevertheless, these findings may be influenced by the type of exercise and mask worn [32,33]. Additionally, the ability to perform vigorous exercise while wearing a mask is an area of concern and controversy [30]. Therefore, it is important to determine whether wearing a surgical or N95 mask during vigorous exercise is well tolerated by individuals who have previously been infected and those who previously have not, in order to provide appropriate recommendations for exercise prescription.

To the best of our knowledge, no study has yet investigated the impact of different mask types on exercise performance in two distinct groups: “previously infected” (PIG) vs. “non-infected” (NIG) groups, particularly during high-intensity, intermittent exercise such as the 5 m shuttle run test (5mSRT). To provide a better understanding of how mask usage affects physical performance, the present study aims to compare the effects of surgical masks (normal masks), N-95 masks and unmasked conditions on the performance of PIG vs. NIG during high-intensity, repeated sprint exercise (5mSRT).

We hypothesize that, (i) compared to the unmasked condition, the performance and the perceived exertion during 5mSRT would be altered using both surgical and N-95 masks, with more pronounced alteration using the N-95, and (ii) compared to non-infected female students, previously infected female students would exhibit greater alterations in the 5 m shuttle run test performance and perceived exertion.

## 2. Materials and Methods

### 2.1. Participants

A group of female physical education students volunteered to take part in this study. To be eligible, participants had to be physically active (e.g., through their studies in the sport science department), with no contraindications for maximal exercise testing (i.e., cardiopulmonary, orthopaedic, or neurological conditions), as confirmed by a medical doctor. They were requested to refrain from consuming caffeine or engaging in strenuous exercise 48 h before all trials.

A participant was classified into the PIG if they had undergone COVID-19 testing at a COVID-19 screening and investigation clinic affiliated with National Guard Health Affairs and tested positive for the virus. The date of infection for each subject corresponds to the day they received an SMS notification from the ‘syhatty’ application identifying them as carriers of the virus. As mandated by the Saudi Arabian government, all candidates in both the PIG and NIG groups received two doses of the COVID-19 vaccine prior to the start of the trial procedure.

Prior to the study, all participants provided signed informed consent, and the study was conducted according to the guidelines of the Declaration of Helsinki and approved by the Ethics Committee of Taif University, Saudi Arabia.

### 2.2. Experimental Design

Participants visited the laboratory on a separate occasion, before the experiment. During this session, they were informed of the study details and requirements; anthropometric measurement and familiarization with the 5 m shuttle run test (5mSRT) were also performed.

Following the familiarization session, participants randomly attended three test sessions (i.e., without mask, with N95 (Figure 1A), and with surgical mask (Figure 1B), separated by at least one week. There is no recommendation to wear a mask in the week before the experiment began or in the weeks in between the test sessions.

Previous research demonstrates that sleep quality and physical activity level have a substantial impact on physical performance [34,35,36]. We monitored these parameters using the IPAQ-SF and the sleep-quality scale to ensure that our results were dependent solely on mask type and whether or not the patient was infected.

At the beginning of each test session, data related to participants’ physical activity and sleep behaviors during the last week were collected (i.e., background data for each test session). In each test session, participants performed the 5mSRT. During the 5mSRT, participants were asked to express their perceived exertion using the Rating of perceived exertion (RPE) scale.

#### 2.2.1. 5 m Shuttle Run Test (5mSRT)

In this test, participants were instructed to perform 6 repetitions of 30 s shuttle sprints with an intermediate recovery of 35 s. Participants focused on performing a maximum distance sprint by going and returning 5 m, then 10 m, then 15 m, then 20 m, etc., for 30 s. After each 30 s repetition, a 35 s recovery was permitted. Distance was reported to the nearest meter. During the recovery phase, participants returned to the starting position in preparation for the subsequent repetition [37]. Participants performed the test in pairs with two investigators to record the results. Based on the distance covered during each repetition, the following indicators were calculated as suggested by Boukhris et al. [38]:

- BD (m) = the greatest distance covered during a 30 s shuttle,
- TD (m) = total distance covered during the six 30 s shuttles,
- FI (%) was calculated as follows:

FI (%) = [(((shuttle 1 + shuttle 2)/2) − ((shuttle 5 + shuttle 6)/2))/((shuttle 1 + shuttle 2)/2)] × 100.

Additionally, the percentage decrement (PD) during the 5mSRT was calculated as follows:


PD (%) = [((BD × number of sprints) − TD)/(BD × number of sprints)] × 100.

#### 2.2.2. Rating of Perceived Exertion (RPE) Scale

After each repetition of 5mSRT, participants gave their subjective RPE score from 0 (very very light) to 10 (very very hard), according to the French version of the CR10 scale, validated by Haddad et al. [39]. Immediately after the end of each 30 s repetition, the participant was shown the RPE scale and asked to report the number from 0 to 10 that best represented their feeling of exertion. The RPE scale is a reliable indicator of physical discomfort, has good psychological measurement characteristics, and correlates closely with several other physiological indicators of fatigue [34]. The following formula was used to calculate the average RPE score during 5mSRT:

$$RPE(AU)=\frac{Sum\;of\;RPE\;scores\;of\;all\;repetitions}{Number\;of\;repetitions}$$

#### 2.2.3. International Physical Activity Questionnaire Short Form (IPAQ-SF)

The data from the IPAQ-SF were added within each item (i.e., vigorous intensity, moderate intensity, and walking), in accordance with the official IPAQ-SF standards to determine the overall amount of time spent engaging in weekly PA [40,41]. By adjusting the reported time for each item by a MET value unique to each PA category, the total weekly PA (MET-minweek-1) was calculated. According to the official IPAQ recommendations for young and middle-aged adults (18–65 years old), MET values established were the initial values (original IPAQ): vigorous PA = 8.0 METs, moderate PA = 4.0 METs, and walking = 3.3 METs. As a fourth and fifth component, respectively, total PA (sum of completed vigorous, moderate, and walking activities) and sitting time were calculated.

#### 2.2.4. Sleep-Related Measures

Participants were asked about their sleeping hours during the previous night and average hours of sleep per week, as well as the subjective quality of their sleep the night before the test on a scale of 0 to 10, where “0” indicates “no sleep,” “5” indicates “some sleep with a few interruptions,” and “10” indicates “deep, uninterrupted sleep” [35].

### 2.3. Statistical Analyses

All statistical tests were processed using STATISTICA 13.0 Software (Stat-Soft, Maisons-Alfort, France). Mean and standard deviation (SD) values were calculated for each variable. Normality of the distribution was confirmed using the Shapiro–Wilks W-test. A two-way analysis of variance (ANOVA) (2 levels [group: with previous infection, without previous infection] × (3 levels [mask conditions: without, N95, FFP2]). When appropriate, post hoc comparisons were performed, and differences were interpreted using a Bonferroni correction. Effect sizes were calculated as partial eta-squared (ηp^2^) to estimate the meaningfulness of significant differences for the normally distributed variables, with values of 0.01, 0.06 and 0.13 representing small, moderate, and large effect sizes, respectively [42]. Significance was accepted for all analyses at the level of *p* < 0.05. Exact *p*-values are provided.

## 3. Results

### 3.1. Participant Characteristics

**Figure 2:**
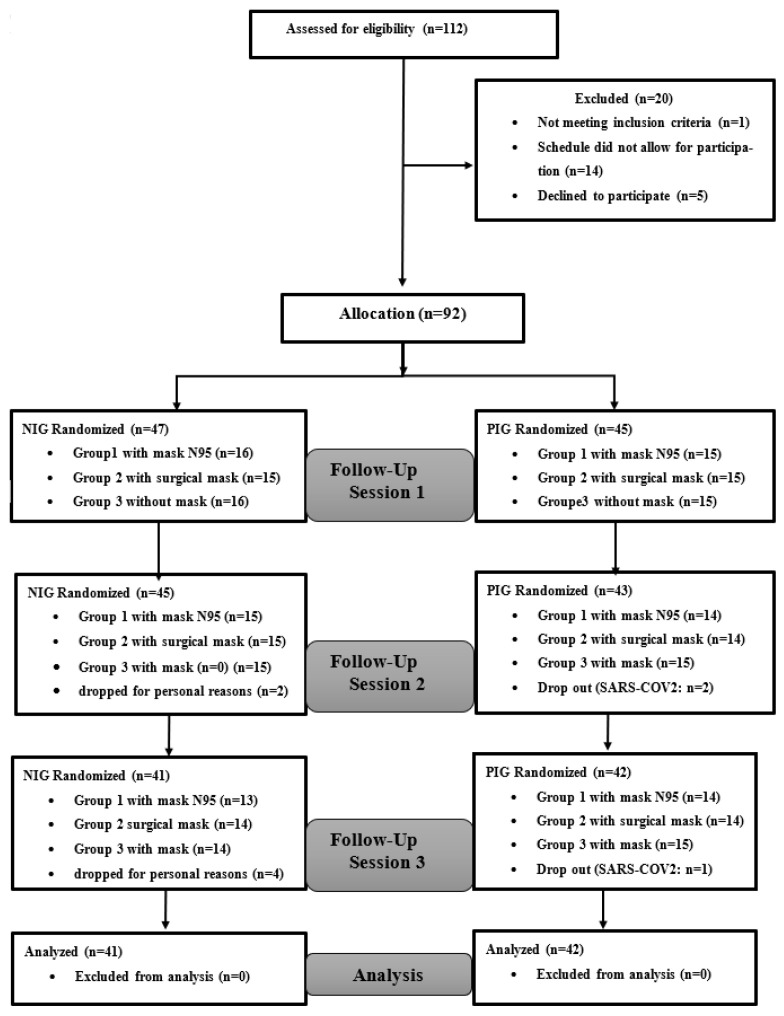
CONSORT flow diagram.

G ∗power 3 software [43] was used to calculate the required sample size. Values for α were set at 0.05 and power at 0.95. Based on the studies of Shaw et al. [21] and Slimani et al. [29] and discussions between the authors, effect size was estimated to be 0.7 (medium effect). The required sample size was twenty. One hundred and twelve participants were screened, and 92 were deemed eligible to participate in this study. Forty-five were allocated to the PIG, and 47 to the NIG. During the experiment, six NIG participants dropped out for personal reasons, and three PIG participants dropped out due to a medical problem (SARS-CoV2). Eighty-three female participants (42 from the PIG and 41 from the NIG; age = 20.02 ± 1.05 years, mean body mass index (BMI) = 21.07 ± 2.1 kg/m^2^) completed the study. All participants in the PIG had developed mild to moderate COVID-19 infections, recovered without hospitalization and/or high-oxygen flow, and had an average diagnosis period of 6.6 ± 4.5 months prior to experimentation. In the results section, data from these 83 participants were included in the final analysis. Figure 2 shows the flowchart of the subject’s screening and participation.

### 3.2. 5 m Shuttle Run Test and RPE 

Table 1 reports mean values for best distance (BD), total distance (TD), fatigue index (FI), percentage decrement (PD), and Rating of perceived exertion scale (RPE) among the different groups, as well as ANOVA results and effect sizes.

As shown in Table 1, the two-way ANOVA indicated no significant main effects of Groups and Mask, and no significant interaction for Groups × Mask for BD, FI, PD and RPE (*p* > 0.05). However, for the TD, the Groups × Mask interaction was significant (F = 3.566, *p* = 0.031, ƞp^2^ = 0.042). The post hoc Bonferroni test showed TD recorded for participants wearing surgical masks was significantly higher in the NIG compared to the PIG (*p* < 0.05). Additionally, for the PIG group, TD was significantly lower with a surgical mask compared to without a mask (*p* < 0.05) and a N95 mask (*p* < 0.05).

### 3.3. Sleep Parameters

Table 2 reports mean values for sleeping hours in the previous night, average sleeping hours in the previous week, and sleep quality among the different groups, as well as ANOVA results and effect sizes.

Results showed sleeping hours during the previous night and sleep quality did not differ significantly between groups (i.e., PIG and NIG) and conditions (i.e., without a mask, with a surgical mask, and with a N95 mask).

However, for average sleep duration over the previous week, sleep duration recorded for participants without a mask was significantly lower in the PIG than the NIG (*p* < 0.05). Contrarywise, sleep duration recorded for participants with a N95 mask was significantly higher in the PIG compared to the NIG (*p* < 0.05). For PIG, sleep duration was significantly higher in participants with the N95 mask compared to participants without a mask (*p* < 0.05).

### 3.4. Physical Activity Behaviors

Table 3 reports mean values for responses to the IPAQ-SF, as well as ANOVA results and effect sizes.

Regarding vigorous intensity, the number of days/week recorded for participants with a surgical mask and N90 mask were significantly higher in the PIG than the NIG (*p* < 0.05). Additionally, for the PIG, the number of days/week were significantly higher in participants with a surgical mask and N95 mask compared to participants without a mask (*p* < 0.05). However, the number of hours/day and MET values did not differ significantly between groups (i.e., infected and non-infected) and conditions (i.e., without a mask, with a surgical mask, and with a N95 mask) (*p* > 0.05).

Regarding moderate intensity, walking, and all PA activities, the number of days/week and hours/week, and MET values did not differ significantly between groups (i.e., PIG and NIG) and conditions (i.e., without a mask, with a surgical mask, and with a N95 mask).

Regarding sitting, the number of hours/day were significantly lower in participants with a N90 mask compared to participants without a mask in the PIG (*p* < 0.05). However, the number of hours/day did not differ significantly between groups (i.e., PIG and NIG) (*p* > 0.05).

## 4. Discussion

The present study investigated the effect of different mask types on high-intensity, intermittent exercise performance in PIG vs. NIG. The first main finding revealed that different mask types did not affect physical performance during the 5mSRT for NIG.

Although these results contradict our initial hypothesis, they are consistent with Shaw et al.’s findings [44]. In their study, the authors found that wearing surgical fabric masks or disposable masks had no appreciable impact on performance during intense exercise in healthy young individuals, as measured by heart rate, arterial oxygen saturation, tissue oxygenation, and perceived exertion during a cycling ergometry test to exhaustion. Moreover, our results are consistent with previous studies showing that healthy individuals tolerate wearing face masks, specially designed to put heavy strain on respiratory muscles, while exercising for several weeks at high intensity without experiencing adverse effects. This suggests using a face mask or respirator during exercise is unlikely to be harmful for healthy people [45]. A review of the literature on the effects of various face masks on the respiratory system during physical activity also indicates that the impact of masks on cardiorespiratory responses to physical activity is minimal, and often imperceptible, even during very intense exercise in healthy individuals [9]. The authors conclude that further research is required to examine the impacts of different masks on the respiratory system during exercise, across diverse populations [9].

However, our findings contrast with those of Driver et al. [46], who reported that wearing cloth face masks increases shortness of breath and discomfort while decreasing exercise time (−14%) and VO2 max (−29%) during high intensity treadmill running exercise. Similarly, wearing surgical and FFP2/N95 face masks led to a decrease in maximum power production during a progressive cycle ergometer test until exhaustion, in healthy individuals [33]. In contrast to the present findings, another study reported that, compared to a no-mask condition, individuals experienced higher RPE and a slightly elevated heart rate while walking at 4 km/h for 6 min on a graduated treadmill (10% rating) while wearing surgical face masks [47].

The second main finding of the present study indicates that, without a mask, there was no significant difference in performance between PIG and NIG, which may indicate a poor association between COVID-19 infection and reduced high-intensity performance in the studied population.

These findings contrast with the study by Steibeis et al. [48], who reported a reduction of <80% of predicted values of ventilatory capacity in 56% of infected young subjects during a cardiopulmonary exercise test (CPET), conducted without masks. The study identified reduced patients’ respiratory quality of life as a risk factor for adverse CPET performance [48]. Furthermore, other studies found that COVID-19 convalescents experienced reduced exercise capacity up to six months after symptom onset, with changes in respiratory efficiency and maximum oxygen consumption (VO2 peak) being the most common anomalies [49,50,51,52,53]. Different studies suggest cardiac and respiratory sequelae, as well as muscle deconditioning, as potential mechanisms underlying the reduction in functional capacity [45,49,54].

The present lack of difference in physical performance between the PIG and NIG in the unmasked condition may be explained by several factors. Firstly, it is important to note our participants were young adults with an average age of 20 ± 1.2; none of them required hospitalization or displayed severe symptoms during their acute phase of COVID-19 infection. Reduced VO2 peak, a well-known risk factor of altered high-intensity running performance, was shown to be associated with dyspnea, hospitalization, and intensive medical care treatment [49,54] with younger subjects and those with lower BMI having higher COVID resistance and less frequent hospitalization [48]. Secondly, it is worth mentioning that all participants were involved, through their academic pursuits in the sport science department, in regular physical activity, which is known to help maintain respiratory and muscular function, even after a COVID-19 infection [19].

However, under the surgical mask condition, TD was significantly reduced in PIG compared to NIG. These findings partially confirm our second hypothesis, as TD was at least 7% lower in PIG in the surgical mask condition during the six 30 s shuttle runs. In general, alterations in the 5mSRT have been previously linked to various factors such as participants’ age, sleep pattern, and physical activity levels [36,38]. The present difference between the PIG and NIG populations using the surgical mask is unlikely to be influenced by any of the abovementioned factors. Age-matched participants showed no difference in terms of 5mSRT performance using the unmasked condition. Additionally, while sleep patterns (quantity and quality) did not differ between both groups in both free-mask and surgical mask conditions, physical performance differed significantly between both groups during the surgical mask condition. Furthermore, MET values for vigorous, walking, moderate, and all PA did not differ between the PIG and NIG across all mask conditions. Differences between both groups were observed in day/week vigorous activity, during surgical and N95 mask conditions. However, while this difference exists under surgical and N95 conditions, 5mSRT performance was only higher in NIG during the surgical mask condition. It seems, therefore, unlikely that physical activity behaviour influenced the TD performance of PIG while wearing a surgical mask.

Overall, our results suggest regular physical activity may be beneficial in preserving respiratory and muscular function in young adults following a COVID-19 infection, as evidenced by the lack of differences between groups in the unmasked condition. However, the cumulative effects of a prior COVID-19 infection plus the use of masks during exercise may negatively impact the performance of PIG, particularly in outcomes requiring sustained high-intensity exercise, such as the TD of the 5mSRT test.

According to present findings, TD was decreased in PIG using a surgical mask compared to no mask and the N95 conditions by 8.23% and 7.17%, respectively.

The lower performance using the surgical mask compared to the unmasked condition is understandable, as wearing a mask is known to make individuals uncomfortable, increase their breathing resistance, and reduce their airflow, which can lead to fatigue and poorer performance [30]. However, the poor performance using the surgical mask compared to the N95 mask was surprising. Previous research indicates that, while N95 offers the greatest protection and the highest filtration effectiveness, it has greater breathing resistance than surgical masks [15,55,56,57].

Li et al. [56] reported that N95 masks had significantly higher rating for perceptions of heat, humidity, breath resistance, and overall discomfort compared to surgical face masks. However, in terms of carbon dioxide inhalation, a more recent study found wearing masks during a moderate physical activity (walking at a speed of 5 km h^–1)^, resulted in an average carbon dioxide concentration of 2875 ppm, with no significant differences between surgical, cloth, or N95 face masks [58]. Importantly, the authors concluded that, according to the existing literature, this concentration could not have a toxicological effect.

Taken together, it seems that using a face mask makes exercising more difficult due to CO_2_ rebreathing; the effect can be mainly attributed to the user’s feeling of greater ventilation rather than a toxicological increase in PaCO_2_ [9]. This assumption can offer insight into the poorer performance recorded in the present study using the surgical mask compared to the unmasked and N95 mask conditions. Nevertheless, it is not possible to provide a firm conclusion on this subject, as the present study lacks measurement of ventilation perception and other physiological parameters.

This assumption is in line with the findings from a recent review on the effects of different face masks and respirators on the respiratory system during physical activity. This review encompassed data from several models, including fabric face covers and surgical masks, N95 respirators, industrial respirators, and high-resistance or high-dead-space applied breathing loads. Overall, the available information from the reviewed studies indicates that, while activity may cause changes in perceived exertion and dyspnea, the effects of face masks on breathing, blood gases, and other physiological parameters during physical activity are minimal, too minimal to be noticed, even during very intense exercise [9].

### Limitations of the Study

This study represents a pioneer effort to compare high-intensity shuttle run performance and perceived exertion using surgical, N95, and no masks among uninfected vs. previously infected populations. However, the study group was small and only included young female students. Therefore, results cannot be extrapolated to the general population. Moreover, additional physiological measures are required to objectively explain the reduction in TD observed only in the surgical mask condition among the PIG.

## 5. Practical Recommendation

In a post-pandemic scenario, even with zero cases and widespread vaccinations, the lasting effects of the COVID-19 pandemic persist, underscoring the ongoing importance of immune system building and preventive measures. The use of various masks is an efficient preventive measure that does not hinder intense, intermittent physical activity for individuals, whether they are previously infected or uninfected. It is crucial for both infected and uninfected individuals to engage in regular physical activity, especially high-intensity intermittent activities, as this contributes to the enhancement of the immune system and overall physical health.

## 6. Conclusions

The present study showed that, under the unmasked condition, there were no differences in 5mSRT performance between previously infected and uninfected active female students enrolled in sport science programs. However, when wearing a surgical mask, PIG covered significantly lower TD compared to NIG. These results suggest post-COVID-19 individuals can maintain physical fitness through regular exercise (i.e., sport science curricula), when exercising without a mask. Additionally, different types of masks did not seem to affect the 5mSRT performance of NIG, whereas PIG exhibited poorer performance when wearing a surgical mask. Accordingly, the impact of different types of face masks on physical performance is suggested to be minimal, particularly in the uninfected population. Nonetheless, this impact remains an active area of research among post-COVID individuals. Future large-scale research is warranted to gain further insight into the impact of different masks on different physical performance indicators and the associated physiological responses among post-COVID individuals.

## Figures and Tables

**Table 1:** Best distance (BD), total distance (TD), fatigue index (FI), percentage decrement (PD) and rating of perceived exertion scale (RPE).

Parameters		Means ± SD			Groups Effect	Mask Effect	Groups × Mask Interaction
		NIG (N = 41)	PIG (N = 42)	All Groups (N = 83)	F(1,81), *p*-Value, ηp^2^	F(1,81), *p*-Value, ηp^2^	F(1,81), *p*-Value, ηp^2^
BD (m)	Without mask	100.4 ± 11.5	100.8 ± 18.9	100.6 ± 15.6	F(1,81) = 1.512, *p =* 0.222, ηp^2^ = 0.018	F(1,81) = 2.072, *p =* 0.129, ηp^2^ = 0.025	F(1,81) = 1.204, *p =* 0.303, ηp^2^ = 0.015
	With surgical mask	101.2 ± 12.3	119.9 ± 97.6	110.6 ± 69.4			
	With mask N95	98.3 ± 12.2	99.2 ± 12.1	98.7 ± 12.1			
TD (m)	Without mask	501.1 ± 73.6	522 ± 106.8	511.6 ± 91.4	F(1,81) = 1.061, *p =* 0.306, ηp^2^ = 0.013	F(1,81) = 0.794, *p =* 0.454, ηp^2^ = 0.010	F(1,81) = 3.566, *p =* 0.031, ηp^2^ = 0.042
	With surgical mask	520.2 ± 95.7	479.2 ± 120.3 ab	499.7 ± 109			
	With mask N95	516 ± 62.7	516.5 ± 77.2 c	516.2 ± 69.8			
FI (%)	Without mask	0.22 ± 0.23	0.18 ± 0.32	0.2 ± 0.28	F(1,81) = 1.997, *p =* 0.161, ηp^2^ = 0.024	F(1,81) = 2.586, *p =* 0.078, ηp^2^ = 0.031	F(1,81) = 2.086, *p =* 0.128, ηp^2^ = 0.025
	With surgical mask	0.2 ± 0.27	0.28 ± 0.29	0.24 ± 0.28			
	With mask N95	0.16 ± 0.09	0.17 ± 0.22	0.17 ± 0.17			
PD (%)	Without mask	0.12 ± 0.07	0.42 ± 1.87	0.27 ± 1.33	F(1,81) = 2.660, *p =* 0.107, ηp^2^ = 0.032	F(1,81) = 0.153, *p =* 0.858, ηp^2^ = 0.002	F(1,81) = 0.156, *p =* 0.856, ηp^2^ = 0.002
	With surgical mask	0.11 ± 0.05	0.36 ± 1.63	0.24 ± 1.14			
	With mask N95	0.12 ± 0.06	0.24 ± 0.84	0.18 ± 0.59			
RPE (a. u.)	Without mask	5.4 ± 1.81	5.11 ± 1.7	5.25 ± 1.76	F(1,81) = 1.524, *p =* 0.221, ηp^2^ = 0.018	F(1,81) = 0.901, *p =* 0.408, ηp^2^ = 0.011	F(1,81) = 0.932, *p =* 0.396, ηp^2^ = 0.011
	With surgical mask	5.18 ± 1.76	5.08 ± 1.6	5.13 ± 1.68			
	With mask N95	5.35 ± 1.77	4.67 ± 1.64	5.01 ± 1.73			

a significantly different from NIG at *p* < 0.05; b significantly different from “Without mask” at *p* < 0.05; c significantly different from “With surgical mask” at *p* < 0.05.

**Table 2:** Sleeping hours previous night, average hours of sleeping previous week and sleep quality for the two groups.

Parameters		Means ± SD			Groups Effect	Mask Effect	Groups × Mask Interaction
		NIG (N = 41)	PIG (N = 42)	All Groups (N = 83)	F(1,81), *p*-Value, ηp^2^	F(1,81), *p*-Value, ηp^2^	F(1,81), *p*-Value, ηp^2^
Sleeping hours last night (hours)	Without mask	4.89 ± 2.99	5.32 ± 3.01	5.11 ± 2.99	F(1,81) = 1.010, *p =* 0.922, ηp^2^ = 0.000	F(1,81) = 0.837, *p =* 0.435, ηp^2^ = 0.010	F(1,81) = 0.451, *p =* 0.638, ηp^2^ = 0.006
	With surgical mask	4.74 ± 2.39	4.58 ± 2.43	4.66 ± 2.39			
	With mask N95	5.02 ± 2.23	4.67 ± 2.46	4.85 ± 2.34			
Average hours of sleeping last week (Hours/week)	Without mask	11.37 ± 11.37	7.68 ± 5.97 a	9.53 ± 9.21	F(1,81) = 0.031, *p =* 0.860, ηp^2^ = 0.000	F(1,81) = 0.548, *p =* 0.579, ηp^2^ = 0.007	F(1,81) = 5.384, *p =* 0.005, ηp^2^ = 0.062
	With surgical mask	8.34 ± 7.36	9.07 ± 6.02	8.71 ± 6.7			
	With mask N95	7.88 ± 6.79 b	12.08 ± 14.89 ab	9.98 ± 11.63			
Sleep quality (a. u.)	Without mask	5.46 ± 2.65	5.77 ± 2.77	5.62 ± 2.69	F(1,81) = 0.176, *p =* 0.676, ηp^2^ = 0.002	F(1,81) = 0.341, *p =* 0.712, ηp^2^ = 0.004	F(1,81) = 0.059, *p =* 0.943, ηp^2^ = 0.001
	With surgical mask	5.66 ± 2.6	5.88 ± 2.43	5.77 ± 2.51			
	With mask N95	5.37 ± 2.78	5.64 ± 2.57	5.5 ± 2.67			

a significantly different from NIG at *p* < 0.05; b significantly different from “Without mask” at *p* < 0.05.

**Table 3:** Responses to the physical activity questionnaire recorded without mask, with surgical mask and with mask N95 for the two groups.

Parameters			Means ± SD			Groups Effect	Mask Effect	Groups × Mask Interaction
			NIG (N = 41)	PIG (N = 42)	All Groups (N = 83)	F(1,81), *p*-Value, ηp^2^	F(1,81), *p*-Value, ηp^2^	F(1,81), *p*-Value, ηp^2^
Vigorous Intensity	(Days/week)	Without mask	0.49 ± 1.45	0.68 ± 1.43	0.59 ± 1.44	F(1,81) = 7.095, *p =* 0.009, ηp^2^ = 0.081	F(1,81) = 0.952, *p =* 0.388, ηp^2^ = 0.012	F(1,81) = 3.616, *p =* 0.029, ηp^2^ = 0.043
		With surgical mask	0.37 ± 0.7	1.22 ± 2.18 ab	0.79 ± 1.65			
		With mask N95	0.29 ± 0.54	1.32 ± 1.72 ab	0.8 ± 1.37			
	(Hours/week)	Without mask	0.29 ± 0.99	0.4 ± 0.65	0.34 ± 0.83	F(1,81) = 3.899, *p =* 0.052, ηp^2^ = 0.046	F(1,81) = 0.647, *p =* 0.525, ηp^2^ = 0.008	F(1,81) = 2.494, *p =* 0.086, ηp^2^ = 0.030
		With surgical mask	0.28 ± 0.64	0.61 ± 1.06	0.45 ± 0.88			
		With mask N95	0.2 ± 0.46	0.73 ± 1.65	0.47 ± 1.24			
	(MET values)	Without mask	311 ± 915	371 ± 877	341 ± 891	F(1,81) = 3.204, *p =* 0.077, ηp^2^ = 0.038	F(1,81) = 1.336, *p =* 0.266, ηp^2^ = 0.016	F(1,81) = 2.663, *p =* 0.073, ηp^2^ = 0.032
		With surgical mask	145 ± 372	1274 ± 4060	709 ± 2904			
		With mask N95	196 ± 355	1270 ± 3905	733 ± 2803			
Moderate Intensity	(Days/week)	Without mask	1.8 ± 1.48	1.71 ± 1.69	1.76 ± 1.58	F(1,81) = 0.419, *p =* 0.520, ηp^2^ = 0.005	F(1,81) = 1.624, *p =* 0.200, ηp^2^ = 0.020	F(1,81) = 2.513, *p =* 0.084, ηp^2^ = 0.030
		With surgical mask	1.51 ± 1.82	1.37 ± 1.75	1.44 ± 1.78			
		With mask N95	1.51 ± 1.53	2.2 ± 2.02	1.85 ± 1.81			
	(Hours/week)	Without mask	0.82 ± 0.8	0.84 ± 0.94	0.83 ± 0.87	F(1,81) = 0.021, *p =* 0.885, ηp^2^ = 0.000	F(1,81) = 0.635, *p =* 0.531, ηp^2^ = 0.008	F(1,81) = 1.103, *p =* 0.334, ηp^2^ = 0.013
		With surgical mask	0.89 ± 0.96	1.06 ± 1.22	0.98 ± 1.09			
		With mask N95	1.03 ± 1.11	0.8 ± 1.07	0.92 ± 1.09			
	(MET values)	Without mask	476 ± 555	439 ± 630	457 ± 590	F(1,81) = 0.120, *p =* 0.730, ηp^2^ = 0.001	F(1,81) = 0.714, *p =* 0.491, ηp^2^ = 0.009	F(1,81) = 0.634, *p =* 0.532, ηp^2^ = 0.008
		With surgical mask	483 ± 634	697 ± 1502	590 ± 1147			
		With mask N95	563 ± 741	565 ± 987	564 ± 866			
Walking	(Days/week)	Without mask	5.12 ± 2.29	5.12 ± 2.44	5.12 ± 2.35	F(1,81) = 0.191, *p =* 0.663, ηp^2^ = 0.002	F(1,81) = 0.450, *p =* 0.638, ηp^2^ = 0.006	F(1,81) = 0.150, *p =* 0.861, ηp^2^ = 0.002
		With surgical mask	5.05 ± 1.97	4.98 ± 2.03	5.01 ± 1.99			
		With mask N95	5 ± 2.03	4.83 ± 2.08	4.91 ± 2.04			
	(Hours/week)	Without mask	2.01 ± 2.3	1.35 ± 1.59	1.68 ± 2	F(1,81) = 3.979, *p =* 0.049, ηp^2^ = 0.047	F(1,81) = 1.249, *p =* 0.290, ηp^2^ = 0.015	F(1,81) = 0.070, *p =* 0.933, ηp^2^ = 0.001
		With surgical mask	2.01 ± 2.2	1.4 ± 1.19	1.7 ± 1.8			
		With mask N95	2.56 ± 3.79	1.68 ± 2.13	2.12 ± 3.1			
	(MET values)	Without mask	2231 ± 2851	1600 ± 2189	1916 ± 2552	F(1,81) = 4.068, *p =* 0.047, ηp^2^ = 0.048	F(1,81) = 0.641, *p =* 0.528, ηp^2^ = 0.008	F(1,81) = 0.257, *p =* 0.774, ηp^2^ = 0.003
		With surgical mask	2202 ± 2881	1397 ± 1437	1799 ± 2310			
		With mask N95	2751 ± 3827	1569 ± 1944	2160 ± 3087			
All PA	(Days/week)	Without mask	5.95 ± 1.73	5.88 ± 1.92	5.91 ± 1.82	F(1,81) = 0.002, *p =* 0.965, ηp^2^ = 0.000	F(1,81) = 0.368, *p =* 0.693, ηp^2^ = 0.005	F(1,81) = 0.899, *p =* 0.409, ηp^2^ = 0.011
		With surgical mask	5.68 ± 1.89	5.76 ± 1.81	5.72 ± 1.84			
		With mask N95	5.78 ± 1.8	6.02 ± 1.8	5.9 ± 1.79			
	(Hours/week)	Without mask	3.12 ± 2.86	2.59 ± 1.94	2.85 ± 2.45	F(1,81) = 0.698, *p =* 0.406, ηp^2^ = 0.009	F(1,81) = 1.543, *p =* 0.217, ηp^2^ = 0.019	F(1,81) = 0.191, *p =* 0.827, ηp^2^ = 0.002
		With surgical mask	3.18 ± 2.76	3.07 ± 2.41	3.13 ± 2.58			
		With mask N95	3.79 ± 3.97	3.21 ± 3.93	3.5 ± 3.94			
	(MET values)	Without mask	3019 ± 3205	2410 ± 2321	2714 ± 2804	F(1,81) = 0.025, *p =* 0.876, ηp^2^ = 0.000	F(1,81) = 1.214, *p =* 0.300, ηp^2^ = 0.015	F(1,81) = 0.816, *p =* 0.444, ηp^2^ = 0.010
		With surgical mask	2830 ± 3064	3368 ± 5702	3099 ± 4540			
		With mask N95	3509 ± 3968	3404 ± 5827	3457 ± 4942			
Sitting	(Hours/day)	Without mask	9.43 ± 4.89	9.2 ± 4.59	9.31 ± 4.72	F(1,81) = 0.008, *p =* 0.930, ηp^2^ = 0.000	F(1,81) = 4.585, *p =* 0.012, ηp^2^ = 0.054	F(1,81) = 0.735, *p =* 0.481, ηp^2^ = 0.009
		With surgical mask	8.65 ± 3.89	8.75 ± 5.55	8.7 ± 4.75			
		With mask N95	8.48 ± 3.99	7.63 ± 4.15 b	8.06 ± 4.06 b			

a significantly different from NIG at *p* < 0.05; b significantly different from “Without mask” at *p* < 0.05.

## Data Availability

Data are available from the first author upon reasonable request.
